# Blood cell gene expression associated with cellular stress defense is modulated by antioxidant-rich food in a randomised controlled clinical trial of male smokers

**DOI:** 10.1186/1741-7015-8-54

**Published:** 2010-09-16

**Authors:** Siv K Bøhn, Mari C Myhrstad, Magne Thoresen, Marit Holden, Anette Karlsen, Siv Haugen Tunheim, Iris Erlund, Mette Svendsen, Ingebjørg Seljeflot, Jan Ø Moskaug, Asim K Duttaroy, Petter Laake, Harald Arnesen, Serena Tonstad, Andrew Collins, Christan A Drevon, Rune Blomhoff

**Affiliations:** 1 Department of Nutrition, Institute of Basic Medical Sciences, Faculty of Medicine, University of Oslo, Norway; 2 Department of Biostatistics, Institute of Basic Medical Sciences, Faculty of Medicine, University of Oslo, Norway; 3 Norwegian Computing Centre, Oslo, Norway; 4 Centre for Occupational and Environmental Medicine, Rikshospitalet-Radiumhospitalet Medical Center, Oslo, Norway; 5 Department of Chronic Disease Prevention, National Institute for Health and Welfare, Helsinki, Finland; 6 Department of Preventive Cardiology, Oslo University Hospital, Ullevål, Oslo, Norway; 7 Centre for Clinical Heart Research, Department of Cardiology, Oslo University Hospital, Ullevål, Oslo, Norway; 8 Department of Biochemistry, Institute of Basic Medical Sciences, Faculty of Medicine, University of Oslo, Norway

## Abstract

**Background:**

Plant-based diets rich in fruit and vegetables can prevent development of several chronic age-related diseases. However, the mechanisms behind this protective effect are not elucidated. We have tested the hypothesis that intake of antioxidant-rich foods can affect groups of genes associated with cellular stress defence in human blood cells. Trial registration number: NCT00520819 http://clinicaltrials.gov.

**Methods:**

In an 8-week dietary intervention study, 102 healthy male smokers were randomised to either a diet rich in various antioxidant-rich foods, a kiwifruit diet (three kiwifruits/d added to the regular diet) or a control group. Blood cell gene expression profiles were obtained from 10 randomly selected individuals of each group. Diet-induced changes on gene expression were compared to controls using a novel application of the gene set enrichment analysis (GSEA) on transcription profiles obtained using Affymetrix HG-U133-Plus 2.0 whole genome arrays.

**Results:**

Changes were observed in the blood cell gene expression profiles in both intervention groups when compared to the control group. Groups of genes involved in regulation of cellular stress defence, such as DNA repair, apoptosis and hypoxia, were significantly upregulated (GSEA, FDR q-values < 5%) by both diets compared to the control group. Genes with common regulatory motifs for aryl hydrocarbon receptor (AhR) and AhR nuclear translocator (AhR/ARNT) were upregulated by both interventions (FDR q-values < 5%). Plasma antioxidant biomarkers (polyphenols/carotenoids) increased in both groups.

**Conclusions:**

The observed changes in the blood cell gene expression profiles suggest that the beneficial effects of a plant-based diet on human health may be mediated through optimization of defence processes.

## Background

The majority of plant chemicals (phytochemicals) are antioxidants. A predominantly plant-based diet reduces the risk for development of many chronic age-related diseases linked to oxidative stress, such as cancer and cardiovascular diseases [1,2]. The hypothesis that the antioxidant abilities of phytochemicals limit oxidative damage caused by reactive oxygen species (ROS) has been tested in large clinical investigations with pharmacological doses of antioxidants (vitamin C, betacarotene and vitamin E). However, these antioxidant supplementations have failed to prevent chronic diseases and in some cases actually worsen the outcome [3,4]. These results are interesting but not contradictory to a biological function of plant food antioxidants. In contrast to antioxidant supplements that contain single or a few antioxidants, plant food contains thousands of different phytochemicals with various antioxidant content and chemical properties. It is possible that a variety of antioxidants at physiological concentrations is required for a beneficial health effect. It is also likely that phytochemicals modulate signal transduction and gene expression related to protective networks via antioxidant as well as through nonantioxidant activities [5,6]. Several phytochemicals can regulate the expression of genes involved in cellular defence via the antioxidant responsive element (ARE/EpRE) by activation of the transcription factor NFE2L2/Nrf2 (nuclear factor (erythroid-derived 2)-like 2/nuclear factor erythroid 2-related factor 2) [7]. Notably, vitamin C, β-carotene and vitamin E are not particularly efficient inducers of NFE2L2 [8,9]. Through evolution, aerobic organisms have acquired inducible networks of genes for protection against damaging effects of ROS [5,6,10] and for repair thereof [11,12]. On the other hand, compelling evidence indicates that ROS is an important mediator, for example, of proper cell signalling, homeostasis control, cell turnover [13]. It has been proposed that oxidative challenges induce adaptive/hormetic responses responsible for the health-promoting effects of physical exercise. Intake of antioxidant supplements during exercise has prevented health-promoting effects of physical exercise in humans [14]. It is possible that ROS as well as plant food substances induce gene expression related to adaptive responses of cellular defence. Removal of ROS by high-dose single antioxidants without the concomitant induction of adaptive/hormetic responses, such as defence and repair processes, may explain the disappointing results of antioxidant supplements on disease prevention. It is also possible that high-dose antioxidants have pro-oxidative effects.

As an approach to identify food with potential beneficial health effects, we have screened more than 3500 different foods for total antioxidant capacity [15]. On the basis of these results, we designed an antioxidant-rich diet including berries, nuts and seeds, spices, certain fruits and vegetables to test the hypothesis that plant food substances induce gene expression related to adaptive responses of cellular stress defence. We also examined whether adding three kiwifruits daily to a regular diet could induce similar effects. In kiwifruit, the majority of measurable total antioxidant content [15] is contributed by one antioxidant (vitamin C) [16].

## Methods

### Study design and intervention

The study followed a randomised group design with 8 weeks of intervention. Healthy male smokers were recruited via an advertisement in a local newspaper.

The inclusion criteria were men between 45 and 75 years of age, smoking at least 5 cigarettes daily and BMI < 35 kg/m^2 ^with stable weight range for the past 6 months. The exclusion criteria were any symptomatic cardiovascular disease (CVD), diabetes (type 1 or type 2), following a vegetarian or near-vegetarian diet, allergy to any items of the intervention or clinically diagnosed disorders, including gastrointestinal diseases. Moreover, participants with a history of serious or unstable medical or psychiatric disorder, current users or those in need of lipid-lowering drug treatment, aspirin or nonsteroidal anti-inflammatory drugs, nutritional supplements or herbs for weight loss within 4 weeks prior to inclusion or participating in a drug trial during the previous 30 days were not included. Participants were included between March 2004 and March 2005. The Easter holiday, summer vacation and Christmas holiday were avoided to prevent possible confounding by seasonal changes in the participants' diets.

The 102 included participants were randomly assigned to the two intervention groups and the control group, with 34 in each group. Randomization was performed in blocks of 12, with group allocation provided in presealed, numbered envelopes. One group received an antioxidant-rich diet while another group received three kiwifruits daily. The antioxidant-rich diet food items were included in exchange for foods in their habitual diet. The food exchange was discussed with the study nutritionist, but the participants were free to select which foods to replace. The kiwifruits were added to the habitual diet. The control subjects continued their habitual diet with a few restrictions (e.g., limitation to coffee intake). In total, 100 participants completed the study (n = 34 in the control group and n = 33 in both the intervention groups). Blood samples were collected at Oslo University Hospital, Ullevål, Norway. Ten individuals from each group were randomly chosen for gene expression analysis of whole-blood RNA using Affymetrix Human Genome U133 Plus 2.0 arrays. All statistics on baseline characteristics and plasma biomarkers in this paper are given for this subgroup only.

The subjects in both intervention groups received food items weekly. In the antioxidant group these included green tea, dog rose juice, cranberry juice, aronia juice, unsweetened bilberry juice, bilberry jam, bilberries, blackberries, strawberries, raspberries, pomegranate, dark blue grapes, Brussels sprouts, broccoli, red cabbage, kale, blue potatoes, tomatoes, dark chocolate, bread with pecan nuts and sunflower seeds, walnuts, olive oil, rosemary, thyme and oregano. The kiwifruit group received a bag of 21 kiwifruits. Weekly amounts of the food items provided to the antioxidant-rich diet group are listed in Table 1. The foods that were not consumed were registered weekly in a custom-made questionnaire. Subjects in the control group were monitored at follow-ups every second week. For whole-blood genome analysis, samples were collected using PAXgene Blood RNA Tubes (QIAGEN, Cat. No. 762115) at the time of randomization and at the end of the intervention period. All investigators performing sample analyses or statistics were blinded until statistical analyses were performed. The study was approved by the regional ethics committee for medical research (REK Sør), and all participants gave their written, informed consent. The study is registered at http://clinicaltrials.gov with Identifier NCT00520819. The trial registry name is 'Oslo Antioxidant Study'.

**Table 1:** Antioxidant-rich food items provided to the antioxidant-rich diet group during the intervention period

Food item	Manufacturer	Food items provided weekly	Total antioxidants provided in foods, mmol/wk
Green java tea	Twinings (London, UK)	7 tea bags	20.86
Juice of rose hips, orange, apple and carrot (Mana yellow)	Tine BA (Oslo, Norway)	1.66 L	42.64
Juice of cranberries, raspberries and grapes (Mana Red)	Tine BA (Oslo, Norway)	1.66 L	10.95
Juice of black chokeberry, bilberries, grapes and cherries (Mana Blå)	Tine BA (Oslo, Norway)	1.66 L	33.18
Bilberry juice (*Vaccinium myrtillus*)	Corona Safteri (Ranheim, Norway)	0.66 L	54.73
Bilberry jam (*Vaccinium myrtillus*)	Heistad (Bergen, Norway)	345 g	10.50
Bilberries (*Vaccinium myrtillus*)	Odd Langdalen Engros	200 g	16.24
Blackberries (*Rubus fruticosus*)	Odd Langdalen Engros	200 g	9.20
Strawberries (*Fragaria × ananassa*)	Odd Langdalen Engros	200 g	4.26
Raspberries (*Rubus idaeus*)	Odd Langdalen Engros	200 g	5.87
Pomegranate (*Punica granatum*)	Odd Langdalen Engros	200 g	3.43
Dark blue grapes (*Vitis sp.*)	Odd Langdalen Engros	200 g	2.23
Brussels sprouts (*Brassica oleracea var. gemmifera*)	Odd Langdalen Engros	200 g	2.01
Broccoli (*Brassica oleracea var. italica*)	Odd Langdalen Engros	200 g	1.93
Red cabbage (*oleracea var. capitata rubra*)	Outdoor cultivar^a^	200 g	3.82
Kale (*Brassica oleracea var. sabellica*)	Outdoor cultivar^a^	200 g	4.71
Blue potatoes (*Solanum tuberosum*, 'Blue congo')	Odd Langdalen Engros (Oslo, Norway)	150 g	0.00
Tomatoes (*Solanum lycopersicum*)	Odd Langdalen Engros (Oslo, Norway)	700 g	2.24
Dark chocolate, 70% cocoa	Kraft Foods (IL, USA)	100 g	11.22
Pecan nuts (*Carya illinoinensis*)^b^	Den lille nøttefrabrikken (Fredrikstad, Norway)	100 g	8.30
Sunflower seeds (*Helianthus annuus*)^b^	Den lille nøttefrabrikken (Fredrikstad, Norway)	100 g	6.31
Walnuts (*Juglans californica*)^b^	Diamond Foods Inc (CA, USA)	200 g	44.48
Extra Virgin Olive Oil (*Olea europaea*)^c^	Ybarra (Toano, Spain)	0.063 L	0.19
Rosemary (*Rosmarinus officinalis*)^c^	Black Boy (Bergen, Norway)	3 g	1.55
Thyme (*Thymus vulgaris*)^c^	Black Boy (Bergen, Norway)	3 g	1.69
Oregano (*Origanum vulgare gracile*)^c^	Black Boy (Bergen, Norway)	3 g	1.90
Total antioxidants per week			304.43

^a^Food items were provided by The Norwegian University of Life Sciences (Ås, Norway).

^b^Food items were provided as ingredients in a bread.

^c^Food items were provided once, at the start of the intervention period.

### Comparison of baseline characteristics and dietary intake

Kruskal-Wallis one-way analysis of variance was used to evaluate whether baseline values or changes during the intervention period differed between the groups. Where significant results were obtained using Kruskal-Wallis, a Mann-Whitney nonparametric test was performed to compare the median values between the groups. Changes during the intervention period were calculated by subtracting the baseline value from the postintervention value. All statistics were performed using SPSS version 14.0. A *P *value of 0.050 or below was considered statistically significant.

### Plasma antioxidants

Quercetin was analysed by high-performance liquid chromatography (HPLC) and electrochemical detection after enzymatic hydrolysis as described elsewhere [17]. Phenolic acids and enterolactone were analysed by gas chromatography-mass spectrometry after enzymatic hydrolysis using a modification of a previously described method [18].

A full description of the methods for plasma carotenoid analyses is included in Additional file 1, document S1. The plasma carotenoids (lutein, zeaxanthin, β-kryptoxanthin, α-carotene, β-carotene and lycopene) were analysed for all groups while the polyphenol (quercetin, phenolic acids and enterolactone) analysis was performed for the antioxidant-rich diet group and control group only, because intake of kiwifruit was not expected to affect changes in these parameters.

### Microarray analysis

**Figure 1:**
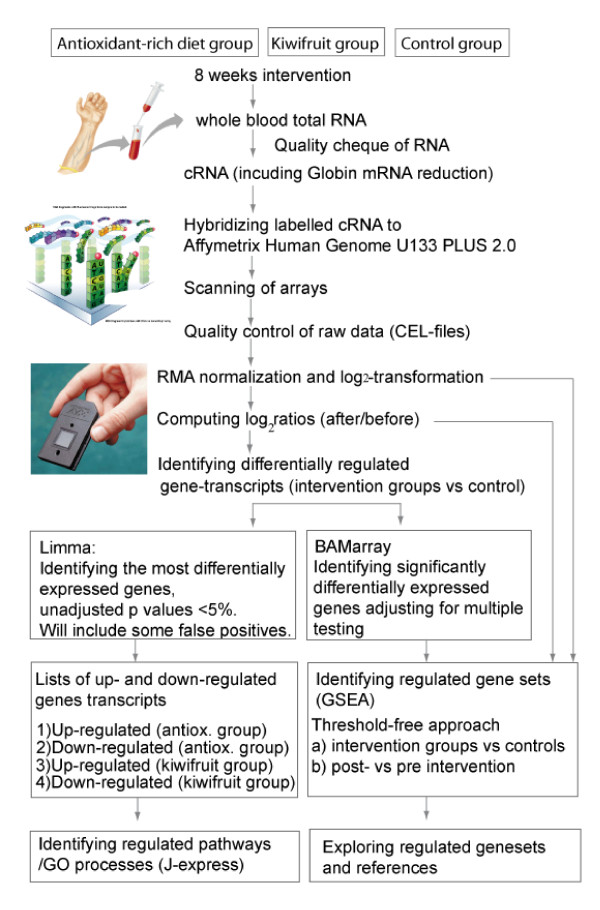
**Overview of the experimental approach and strategy for data analyses**. (Images courtesy of Affymetrix Inc.).

An overview of the experimental approach and strategy for data analyses is presented in Figure 1. RNA from whole blood was isolated according to the PAX kit manufacturer, including the optional on-column DNase digestion. All samples in the subgroup used for microarray analysis (n = 30) had excellent RNA integrity as judged by Bioanalyzer and sufficient yield (> 10 μg RNA). Affymetrix one-cycle gene expression protocol was performed according to Affymetrix (Santa Clara, CA) by including the step of globin transcription reduction (GeneChip globin reduction, Affymetrix). The globin reduction oligos (PNA oligos) were purchased from Applied Biosystems. All other reagents were purchased from Affymetrix. Affymetrix Human Genome U133 Plus 2.0 arrays were used to obtain whole-blood RNA expression profiles. Labeled cRNA was analysed by Bioanalyzer and Nanodrop before fragmentation and hybridization to the arrays. To verify that reverse transcription of globin mRNA was successfully blocked, RNA was analysed by gel electrophoresis (data not shown).

### Microarray data analysis

Minimum Information About a Microarray Experiment (MIAME) standards http://www.mged.org/Workgroups/MIAME/miame.html were followed in the analysis and storage of data. The raw data are available at the ArrayExpress at http://www.ebi.ac.uk/microarray-as/ae/ at accession number E-MEXP-2030. Arrays were scanned using GeneChip Scanner 3000 7G (Affymetrix). Image analysis was performed using GeneChip Operating Software 1.3 (Affymetrix). Libraries from http://www.bioconductor.org (e.g., affy, simpleaffy, affyPLM, arrayQuality) implemented in the MADMAX quality control pipeline https://madmax.bioinformatics.nl were used to assess the quality of the arrays in the experiment. Arrays from one subject in the kiwifruit group were removed due to evident hybridization problems. For every gene, the change in gene expression during the intervention were obtained by calculating log_2 _ratios between the before and after intensities using Robust Multichip Average (RMA) normalised intensities. The intervention groups were then compared to the controls with regard to this ratio. Percentage change for each gene and maximum fold induction and reduction were calculated.

Probe set annotation was last updated on 12 March 2009 via the NetAffx on the Affymetrix web site http://www.affymetrix.com/analysis/index.affx. The basic local alignment search tool (BLAST) for aligning two nucleotide sequences was utilised for identification of the probe sets target sequences, and the BLAST database was used for identification of sequences of the differentially expressed genes.

### Differentially expressed genes

A specialised Bayesian analysis of variance (ANOVA) method for microarrays, BAMarray http://www.BAMarray.com, which accounts for the problem of multiple testing, was used to find significantly differentially expressed genes [19].

### Pathway analysis

To create lists of differentially regulated gene transcripts for identification of biological processes, we applied a moderated *t*-test using R (version 2.6.1) and Linear Models for Microarray data [20] (Limma package from http://www.Bioconductor.org) on the RMA-normalised log_2 _ratios of the three groups (control group, antioxidant-rich diet group and kiwifruit group). The differentially expressed genes were divided into up- and downregulated gene transcripts for both intervention groups, thus producing four different lists. The threshold for including gene transcripts in the lists was an unadjusted/nominal *P *value (*P*_nom_) of < 5%.

For further downstream analysis, we used J-express provided by http://www.molmine.com. Fisher's exact test was used to explore whether the distribution of processes or pathways as defined by the Gene Ontology consortium http://www.geneontology.org in a subset of genes may be expected by chance when compared with a reference. Files required to run the application were downloaded from http://www.geneontology.org according to the instructions. The gene abbreviations used were those of the (Human Genome Organisation) HUGO gene nomenclature committee.

### Gene set enrichment analysis (GSEA)

GSEA was used to identify whether an *a priori *defined set of genes (e.g., a pathway) shows concordant statistically significant differences between two biological states (e.g., diet intervention vs. control) [21]. Collections of gene sets were obtained using a gene set browser from the Broad Institute website http://www.broad.mit.edu/gsea/. This browser searches a number of publicly available sources (e.g., Biocarta, Kegg) where genes are grouped if they belong to the same pathway and share ontology terms or clinical phenotypes. Thus, it is possible to define specific gene sets on the basis of a particular parameter of interest. We created 10 collections of gene sets using the following keywords stepwise: 'DNA AND repair'. Apoptosis', 'cytokine*', 'inflammation', interleukin*', 'immune* AND response', 'Hypoxi*', 'Stress', 'Stress AND response' and 'Oxidative AND stress'. The large, predefined gene set collection C3-TFT (transcription factor targets) version 2.5 from http://www.broad.mit.edu/gsea/[22] was also used. The C3 TFT collection consists of gene sets that contain genes that share a transcription factor binding site defined in the TRANSFAC (version 7.4, http://www.gene-regulation.com/).

GSEA was performed using J-express according to the description on the J-express manual. The gene matrix was collapsed by selecting the maximum probes. T statistics were used as the scoring method, the number of permutations was set to 1000 and gene sets with less than 10 genes or more than 500 genes were excluded from the analysis. False discovery rate (FDR) *q *values < 5% were used as criteria for significantly enriched gene sets. Leading edge genes contributing to the significance of the regulated gene sets associated with DNA and repair were analysed using MetaCore from GeneGo Inc. http://www.genego.com/, an integrated software suite for functional analysis of biological experimental data.

## Results

### Participant characteristics

**Figure 2:**
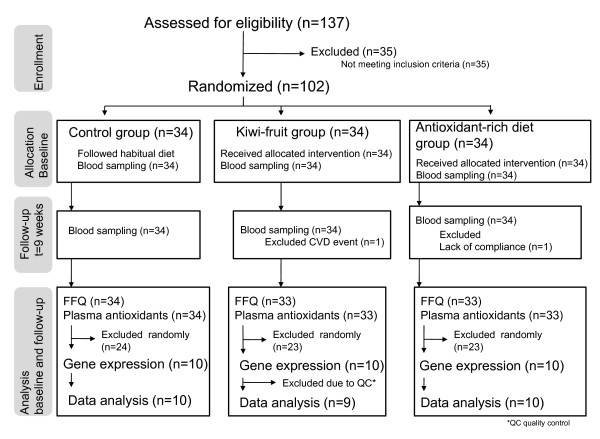
**Participant flow**.

Of the 100 participants who completed the study (n = 34 in the control group and n = 33 in both the intervention groups), 10 individuals from each group were randomly chosen for microarray analysis (Figure 2).

Participants in the microarray subgroups of the antioxidant-rich diet (n = 10), kiwifruit (n = 9) and control groups (n = 10) were not significantly different at baseline with respect to age, BMI and the number of cigarettes smoked per day (Kruskal-Wallis) (Table 2). There were no significant differences between the three groups at baseline concerning intake of energy, alcohol, total fat, polyunsaturated fatty acids (PUFA), monounsaturated fatty acids (MUFA), saturated fatty acids (SFA), protein or carbohydrates (Kruskal-Wallis; data not shown).

**Table 2:** Baseline characteristics

	Control group (n = 10)		Antioxidant-rich diet group (n = 10)		Kiwifruit group (n = 9)		*P *value
Age (yr)	56.0	(49.0-61.0)	59.0	(53.0-63.0)	57.0	(53.0-62.0)	0.3
Cigarettes (n/d)	18.0	(10.0-20.0)	11.6	(5.0-20.0)	18.0	(10.0-20.0)	0.5
BMI (kg/m^2^)	25.6	(22.1-28.6)	25.6	(22.9-26.2)	23.5	(22.3-28.4)	0.8

Group median with 95% confidence interval. A Kruskal-Wallis test was used to compare the groups.

### Plasma antioxidants

Plasma antioxidants increased in both intervention subgroups (Tables 3 and 4) compared to controls, indicating successful dietary intervention. The significant median increase in plasma concentrations in the antioxidant-rich diet group were as follows: lutein (37%), α-carotene (221%), β-carotene (50%), vanillic acid (30%), protocatechuic acid (36%), 3,4-dihydroxyphenyl acetic acid (DOPAC) (19%) and gallic acid (94%). The following polyphenols showed a trend for increase: quercetin (105%, *P *= 0.11) homovanillic acid (20%, *P *= 0.08) and *p*-coumaric acid (68%, *P *= 0.08). A significant decrease was observed for zeaxanthin (-22%). In the kiwifruit group, increases that were significantly different from control group were lutein (37% increase) and β-kryptoxanthin (-33% decrease).

**Table 3:** Plasma carotenoids (nmol/L) and biomarkers of carotenoid-rich dietary items

	Control group (n = 10)				Antioxidant-rich diet group (n = 10)				Kiwifruit group (n = 9)				
	
	Baseline		Change		Baseline		Change		Baseline		Change		*P*^a^
Lutein	0.13	(0.08-0.18)	0.00	(-0.03-0.04)	0.14	(0.11-0.20)	0.05	(0.00-0.12)	0.14	(0.08-0.22)	0.05	(-0.02-0.07)	0.07^b^
Zeaxanthin	0.03	(0.02-0.04)	0.00	(-0.01-0.01)	0.04	(0.02-0.06)	-0.01	(-0.03-0.00)	0.03	(0.02-0.05)	0.00	(-0.01-0.01)	0.03^c^
β-cryptoxanthin	0.07	(0.03-0.16)	0.00	(-0.03-0.01)	0.12	(0.06-0.23)	-0.05	(-0.13-0.01)	0.08	(0.06-0.12)	-0.03	(-0.04-0.00)	0.10
α-carotene	0.06	(0.03-0.09)	-0.01	(-0.04-0.00)	0.03	(0.02-0.11)	0.07	(0.00-0.18)	0.07	(0.06-0.08)	-0.01	(-0.02-0.04)	0.01^c^
β-carotene	0.26	(0.22-0.38)	-0.04	(-0.26-0.05)	0.16	(0.11-0.66)	0.08	(-0.01-0.17)	0.36	(0.13-0.52)	-0.05	(-0.11-0.07)	0.02^c^
Lycopene	0.50	(0.20-0.71)	-0.01	(-0.12-0.34)	0.58	(0.30-0.88)	-0.10	(-0.41-0.14)	0.57	(0.41-0.68)	0.04	(-0.04-0.30)	0.11

Group median with 95% confidence interval.

^a^Kruskal-Wallis test was used to compare the changes between the groups. A Mann-Whitney test was used as post hoc test.

^b^Change during intervention is significantly different between the kiwifruit group and controls.

^c^Significant difference between the antioxidant-rich diet group and controls. Baseline values were not significantly different between the groups (*P *values not shown).

**Table 4:** Plasma polyphenol concentrations (nmol/L).

	Control (n = 10)				Antioxidant-rich diet (n = 10)				
	Baseline		Change		Baseline		Change		*P *value^a^
Paraxanthin	3.2	(0.4-5.9)	-0.9	(-3.6-0.6)	2.0	(0.0-7.1)	-0.5	(-3.3-0.2)	0.85
Quercetin	24.5	(2.0-67.8)	3.1	(-65.8-22.2)	21.2	(2.0-54.6)	22.2	(-0.9-193.6)	0.11
3-hydroxyphenyl acetic acid	85.0	(28.9-605.7)	15.6	(-309.4-91.4)	92.3	(26.1-294.4)	50.4	(-90.5-735.4)	0.51
4-hydroxyphenyl acetic acid	500.6	(376.1-1038.2)	-37.5	(-254.1-353.0)	587.9	(400.5-923.6)	-106.4	(-497.7-225.9)	0.68
Vanillic acid	128.9	(99.0-679.5)	-41.0	(-153.3-42.1)	118.7	(73.8-318.4)	64.2	(-3.8-552.0)	0.02
Protocatechuic acid	112.5	(54.3-327.5)	-5.8	(-71.4-23.4)	88.7	(61.8-156.1)	31.9	(6.6-53.8)	0.03*
Homovanillic acid	95.6	(77.9-279.1)	-1.4	(-187.5-21.3)	87.3	(77.6-144.0)	17.3	(-21.6-255.5)	0.08
3,4-hydroxyphenyl acetic acid	150.1	(92.6-199.8)	-5.5	(-89.4-24.8)	158.4	(107.8-235.8)	31.4	(-54.1-102.7)	0.12
Gallic acid	30.4	(16.0-63.3)	-4.6	(-28.9-20.7)	21.7	(15.5-29.8)	20.3	(0.8-39.8)	0.01*
*p*-coumaric acid	20.8	(11.9-110.2)	-6.5	(-65.0-1.8)	15.3	(5.6-36.1)	10.4	(-15.4-83.1)	0.08
Caffeic acid	203.1	(32.7-656.5)	-29.2	(-255.4-24.7)	97.6	(36.8-193.5)	-10.4	(-112.0-232.4)	0.31
Enterolactone	18.1	(4.8-39.2)	9.1	(-2.6-55.1)	13.0	(6.2-50.6)	10.5	(-4.7-40.6)	1.00

Biomarkers of polyphenol intake.

Group median with 95% confidence interval.

^a^A Mann-Whitney test was used to compare the change in the antioxidant-rich diet group compared to controls.

*Significant *P *< 0.05. Baseline values were not significantly different between the groups. *P *values not shown.

### Gene expression profiling

Figure 1 describes the experimental approach and the strategy for data analyses. Changes in gene expression in the intervention groups during the 8-week intervention were compared to the control group. We identified significantly differentially expressed genes and biological processes and pathways modulated by the dietary interventions. The methods used are based on different approaches to the statistical analysis as well as to how the gene transcripts are grouped. While GSEA was used to test the specific hypothesis that groups of genes involved in stress and defence processes were changed during the intervention period, the gene ontology (GO) analysis on regulated gene lists is a more descriptive method.

### Identification of significantly differentially expressed genes

By applying BAMarray, we identified 44 gene transcripts as differentially expressed in the antioxidant-rich diet group as compared to the control group (adjusted for multiple testing). In the kiwifruit group, nine gene transcripts were significantly altered compared to the control group. The maximum induction of gene transcripts in the antioxidant-rich diet group was 77%, whereas the kiwifruit group had a maximum increase of 37%. The maximum reduction was 34% in the antioxidant-rich diet group and 23% in the kiwifruit group.

Twenty-six of the differentially expressed genes in the antioxidant-rich diet group were identified as encoding proteins with known chromosomal location and function (Table 5). The identified gene transcripts are known to be associated with processes such as signal transduction, lipid metabolism, transcriptional regulation, intracellular transport, cytoskeleton organization and inflammatory response. Two of the downregulated gene transcripts, hormone-sensitive lipase (LIPE) and glycerol kinase 2 (GK2), are targets for the nuclear receptor, peroxisome proliferator-activated receptor-γ (PPARγ) and are involved in lipid metabolism. In addition, the nuclear receptor subfamily 0 (NR0B1) known as a PPARγ corepressor was also downregulated compared to the control group. Several of the downregulated transcripts represent genes involved in signal transduction, including the two G protein-coupled receptors: bitter taste receptor (TAS2R16) and olfactory receptor 2 (OR2F1). The probe ID 1567015_at which was significantly downregulated is annotated NFE2L2 nuclear factor (erythroid-derived 2)-like 2 by the NetAffx from Affymetrix. However, we performed a BLAST aligning approach and found this probe ID to be wrongly annotated. Five of the nine gene transcripts that were significantly altered in the kiwifruit group are annotated with known functions (Table 6) and are associated with signal transduction (G protein-coupled receptor 173 (GPR173) (down), creatine kinase muscle (CKM) (down), catenin-δ (CTNND1) (up)), ion transport (Na^+^/H^+ ^exchanger domain containing 1 (NHEDC1 (up)) and immune functions (immunoglobulin κ light chain variable region (NTN3) (down)).

**Table 5:** Genes that were significantly upregulated (positive z-score) and downregulated (negative z-score) in the antioxidant-rich diet group when compared to controls.

Probe id	*z*-score	Gene Symbol	Gene title
208526_at	-4.4	OR2F1	Olfactory receptor, family 2, subfamily F, member 1
221444_at	-4.1	TAS2R16	Taste receptor, type 2, member 16
1559244_at	-4.0	FMN2	Formin 2
1553706_at	-4.0	HTRA4	HtrA serine peptidase 4
1553652_a_at	-3.8	C18orf54	Chromosome 18 open reading frame 54
1559270_at	-3.6	ZFHX4	Zinc finger homeobox 4
227401_at	-3.5	IL17D	Interleukin 17D
206644_at	-3.4	NR0B1	Nuclear receptor subfamily 0, group B, member 1
229731_at	-3.3	FOXS1	Forkhead box S1
233897_at	-3.3	FEZF2	FEZ family zinc finger 2
233305_at	-3.3	NECAB1	N-terminal EF-hand calcium binding protein 1
213855_s_at	-3.2	LIPE	Lipase, hormone-sensitive
207817_at	-3.1	IFNW1	Interferon, omega 1
203059_s_at	-3.1	PAPSS2	3'-phosphoadenosine 5'-phosphosulfate synthase 2
205893_at	-2.9	NLGN1	Neuroligin 1
222041_at	-2.8	DPH1	DPH1 homolog (*Saccharomyces cerevisiae*)///candidate tumor suppressor in ovarian cancer 2
215430_at	-2.8	GK2	Glycerol kinase 2
202855_s_at	6.6	SLC16A3	Solute carrier family 16, member 3 (monocarboxylic acid transporter 4)
202856_s_at	4.0	SLC16A3	Solute carrier family 16, member 3 (monocarboxylic acid transporter 4)
218505_at	3.7	WDR59	WD repeat domain 59
34726_at	3.5	CACNB3	Calcium channel, voltage-dependent, beta 3 subunit
211079_s_at	3.4	DYRK1A	Dual-specificity tyrosine-(Y)-phosphorylation regulated kinase 1A
57163_at	3.1	ELOVL1	Elongation of very long chain fatty acids (FEN1/Elo2, SUR4/Elo3, yeast)-like 1
225466_at	3.1	PATL1	Protein associated with topoisomerase II homolog 1 (yeast)
1569701_at	3.0	PER3	CDNA FLJ58931 complete cds, highly similar to Period circadian protein 3

**Table 6:** Genes that were significantly regulated in the kiwifruit group when compared to controls.

Probe ID	*z*-score	Gene Symbol	Gene Title
204810_s_at	-3.3	CKM	Creatine kinase muscle
217034_at	-4.2	NTN3	Immunoglobulin-κ light chain variable region (IGKV gene), clone 25
221299_at	-3.9	GPR173	G protein-coupled receptor 173
1553633_s_at	4.3	NHEDC1	Na^+^/H^+ ^exchanger domain containing 1
1557944_s_at	3.8	CTNND1	Catenin (cadherin-associated protein)-δ 1

Positive *z*-score means upregulation in the kiwifruit group whereas negative *z*-score means downregulation.

### Identification of differentially regulated gene sets by GSEA

GSEA was used to test the hypothesis that groups of genes involved in stress and defence processes were changed during the intervention period by comparing the two interventions to the control. A substantial number of 'stress'-related gene sets in the defined collections, as described in Methods section, were significantly upregulated in the intervention groups (Table 7).

**Table 7:** Regulation of stress relevant gene sets comparing the intervention groups to the controls with regard to changes in gene expression (FDR ≤ 5%)

		Number of Upregulated Gene Sets			
		Antioxidant-Rich Diet Group		Kiwifruit Group	
		
GSEA Gene Set Collections^1^	Number of Gene Sets in Collection^2^	Up	Down	Up	Down
DNA and repair	61	15	0	13	0
Hypoxia*	33	4	0	1	0
Apoptosis	206	11	0	4	0
Cytokine*	164	1	0	0	0
Interleukin*	98	1	0	1	0
Immune* and response	62	1	0	1	1
Inflammation	7	1	0	0	0
Stress	91	6	0	0	0
Stress and response	51	4	0	0	0
Oxidative and stress	32	2	0	0	0
C3TFT^3^	582	13	4	3	0

^1^Collections of gene sets were obtained using a gene set browser from the Broad Institute web site http://www.broad.mit.edu/gsea/ defined by the listed keywords. For some keywords we used truncated search terms as indicated by *.

^2^Number of gene sets passing the gene set size filter.

^3^The C3 TFT gene set collection consists of gene sets grouped by common transcription factor binding sites.

The 'DNA and repair' collection, consisting of 61 gene sets, is the most convincing significantly upregulated gene set collection in both intervention groups as compared to the control group, with 15 and 13 gene sets upregulated in the antioxidant-rich diet group and kiwifruit group, respectively. Ten of these were overlapping between the intervention groups. As shown in Additional files 2. 3, 4, Figures S1-S3, the leading edge genes contributing to the significance of these gene sets are involved in different aspects of DNA repair such as nucleotide excision repair, mismatch repair and double-stranded break repair. Gene set collections related to hypoxia, and apoptosis were upregulated in both intervention groups with larger effects, i.e., a higher number of regulated gene sets in the antioxidant-rich diet group. Only one gene set in each of the three immune-related collections was upregulated in the antioxidant-rich diet group, while two immune-related gene sets were upregulated in the kiwifruit group. One gene set related to immunoglobulin secretion was also found downregulated in the kiwifruit group. The 'stress', 'stress and response' and 'oxidative stress' collections were significantly upregulated in the antioxidant-rich diet group but not in the kiwifruit group. An overview of the regulated gene sets in each collection is provided as Additional files 5, 6, Tables S1 and S2, including a brief description of the differentially regulated gene sets with references. Additional information on the gene sets can also be obtained at http://www.broadinstitute.org/gsea/.

Paired GSEA tests comparing pre- and postintervention values within each group confirmed that the 'stress'-related gene sets were significantly upregulated in the intervention groups (Table 8). No gene sets related to DNA repair, hypoxia, stress response or oxidative stress were upregulated in the control group, whereas some immune-related gene sets were significantly modulated (both up- and downregulated).

**Table 8:** Regulation of stress relevant gene sets comparing pre and post intervention gene expression within each group (FDR ≤ 5%)

GSEA Gene Set Collections	Number of up regulated Gene Sets					
	
	Control Group		Kiwifruit Group		Antioxidant-Rich Diet Group	
	
	Pre	Post	Pre	Post	Pre	Post
DNA and repair (61)	2	0	0	10	0	20
Hypoxia* (33)	0	0	0	2	0	3
Apoptosis (209)	1	3	0	21	0	30
Cytokine* (164)	0	3	0	9	0	8
Interleukin* (98)	1	1	0	10	0	2
Immune* and response (62)	0	2	0	11	0	6
Inflammation (7)	0	0	0	2	0	0
Stress and response (51)	0	1	0	0	0	8
Stress (91)	0	0	0	0	0	11
Oxidative and stress (32)	0	0	0	0	0	1
C3TFT (582)	0	0	1	0	0	45

^1^Collections of gene sets were obtained using a gene set browser from the Broad Institute website http://www.broad.mit.edu/gsea/ defined by the listed keywords. For some keywords we used truncated search terms as indicated by *.

^2^Number of gene sets in each collection passing the gene set size filter is given in parentheses.

^3^The C3 TFT gene set collection consists of gene sets grouped by common transcription factor binding sites.

To explore which regulatory mechanisms may be involved in the gene regulation induced by the two diets, we performed a GSEA analysis to test whether groups of genes with common regulatory motifs were differentially regulated. We found 13 significantly upregulated C3 transcription factor targets (TFT) gene sets in the antioxidant-rich diet group, of which six were known transcription factors (Additional file 7, Table S3). Two of these are genes containing the promoter motive for Yin-Yang transcription factor (YY1). The other represents genes with promoter motifs for GA binding protein transcription factor (GABP)/nuclear respiratory factor 2 (NRF2), member of the E-26 (ETS) oncogene family (ELK1) and aryl hydrocarbon receptor (AhR). Four C3 TFT gene sets were downregulated in the antioxidant-rich diet group. Two of these are grouped by similar promoter motives for known transcription factors; cutlike 1 (CUTL1) and RE1-silencing transcription factor (REST). Three C3 TFT gene sets were upregulated in the kiwifruit group. Two of these are grouped by similar promoter motives for known transcription factors: the AhR and hypoxia-inducible factor 1 (HIF1A), respectively. No C3 TFT gene sets were downregulated in the kiwifruit group.

### Identification of regulated biological processes among the differentially expressed transcripts

Up- and downregulated gene transcript lists obtained by Limma, as described in the Methods section, were analysed separately in the downstream applications looking for patterns with regard to biological processes or pathways. A total of 569 and 577 gene transcripts were upregulated by the antioxidant-rich diet intervention and by the kiwifruit intervention, respectively, as compared to the control group (Additional files 8 and 9, Tables S4 and S5). Seventy of these were upregulated in both intervention groups. Compared to the control group, 1162 and 887 gene transcripts were downregulated by the antioxidant-rich diet intervention and by the kiwifruit intervention, respectively (Additional files 10 and 11, Tables S6 and S7). The number of downregulated gene transcripts common for the two interventions was 208.

A paired moderate *t*-test (Limma) was also performed on the log_2 _values of the control, kiwifruit-rich, and antioxidant-rich diet groups separately. A total of 2415 gene transcripts were differentially modulated by the intervention in the antioxidant-rich diet group (1261 downregulated and 1151 upregulated), 2034 were modulated in the kiwifruit group (1064 downregulated and 970 upregulated), whereas the control group had 1622 regulated gene transcripts (700 downregulated and 922 upregulated) (*p*_nom _< 5%) (data not shown). These lists confirmed that more gene transcripts were regulated by the interventions compared to the control group. The lists obtained from the paired analyses were not used for downstream analysis.

To explore whether a biological process is enriched among the differentially regulated gene transcripts, the GO analysis in J-express was used. This tool tests whether the distribution of a biological process in a list of regulated gene transcripts may be expected when compared to a reference (all GO processes associated with the HG-133 plus 2 chip). A number of biological processes were significantly enriched in the lists of up- and downregulated gene transcripts for both intervention groups (Additional files 12, 13, 14, 15, Tables S8-S11). Biological processes significantly enriched are listed hierarchically. Processes with less than two genes are not included in the table. One gene transcript may map to several biological processes. Biological processes related to 'response to stress', such as DNA repair and defence responses, were significantly enriched in the list of upregulated gene transcripts in both intervention groups and were not found in the downregulated lists. Immune-related processes and processes relevant for regulation of apoptosis were enriched in both up- and downregulated gene transcript lists for both intervention groups.

## Discussion

To our knowledge, this human intervention study is the first in which effects of a plant-based diet have been measured in blood cells using whole genome microarray technology. We have applied a novel application of the GSEA method to test the hypothesis that intake of antioxidant-rich foods has an effect on groups of genes associated with cellular stress defence in human blood cells and present data supporting this view.

**Figure 3:**
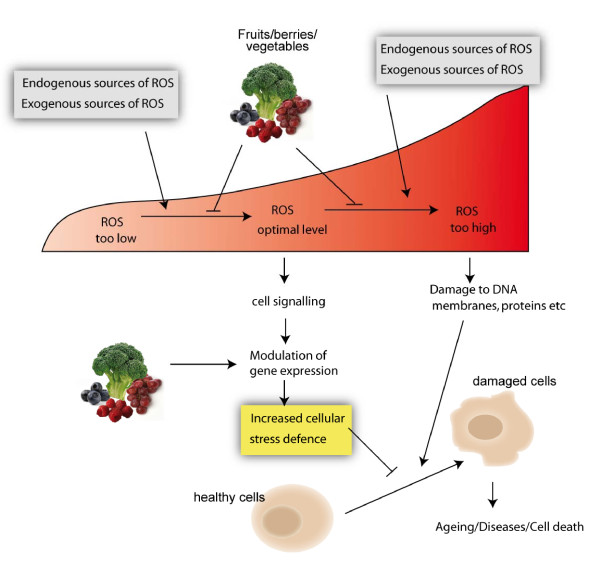
**Hypothetical model suggesting how plant food phytochemicals influence protective cellular defence systems (yellow box) in a similar manner as an optimal level of reactive oxygen species (ROS)**. It is possible that phytochemicals work by modulating ROS levels while regulating inducible defence systems.

It is widely accepted that accumulation of molecular and cellular damage, together with progressive failure of maintenance and repair processes, is associated with aging and that plant-based diets protect against age-related diseases [2,23]. Induction of defence pathways by phytochemicals has been proposed to explain the beneficial effects of a plant-based diet [24]. Both energy restriction and regular physical activity are thought to improve life expectancy by inducing adaptive/hormetic responses that protect against molecular damage and subsequent premature aging [25-28]. It is possible that components of plant foods may promote health effects and longevity through similar mechanisms (Figure 3).

One of the few natural compounds that can increase lifespan in animals is resveratrol, a phenolic compound found mainly in the skin of grapes [29]. The antioxidant-rich diet used in this intervention study contains not only dietary sources of resveratrol but also thousands of other plant compounds with potential bioefficiency.

Upregulation of nuclear genes related to DNA repair, metabolism, apoptosis and 'stress' response has been reported in several studies in association with longevity [30]. For instance, human centenarians are observed to have higher activity levels of poly(ADP-ribose) polymerase-1, which is a key player in the immediate cellular response to stress-induced DNA damage [31]. The transcripts associated with such mechanisms that are upregulated by both the antioxidant-rich diet and the kiwifruit diet may therefore be similarly important for cellular stress defence and maintenance. In agreement with our findings, a moderate and significant upregulation of DNA repair capacity in lymphocytes has been found after a 3-week intervention with cooked carrots [32], and several DNA repair genes were upregulated following a flavonoid-rich diet for 4 weeks [33]. Thus, we suggest that upregulation of genes involved in different DNA repair pathways may explain the observed increase in lymphocyte DNA repair capacity induced by plant-enriched diets. The upregulation of target genes for transcription factors involved in stress responses in our study also offers some potential mechanistic explanations behind the beneficial health effects of plant-based diets. Genes sharing the regulatory xenobiotic response element (XRE) for the aryl hydrocarbon receptor (AHR)/AhR nuclear translocator (ARNT) were upregulated in both intervention groups. Phytochemicals may act as ligands for AHR [34]. Ligand activation of AHR results in dimerisation with ARNT with subsequent binding to XRE. Genes controlled by XRE are involved in Phase I and Phase II reactions whose primary function is to inactivate and eliminate harmful xenobiotic substances. We also found genes with the regulatory motif for hypoxia-inducible factor 1 A (HIF1A) upregulated in the kiwifruit group. HIF1A controls the hypoxic response occurring at low oxygen tension. Under hypoxia, HIF1A dimerises with ARNT, allowing translocation into the nucleus for induction of gene expression. HIF1A target genes are involved in stress and defence responses [35]. The hypoxic response is also important for proper immune function [36]. Our data therefore support the hypotheses that intake of a diet rich in antioxidants induces expression of detoxification enzymes and proteins involved in defence and stress responses.

Target genes for nuclear respiratory factor 2 (GABP/NRF2) were upregulated in the antioxidant-rich diet group. Nuclear respiratory factors enhance the expression of nuclear genes involved in mitochondrial function and biogenesis, such as the respiratory subunits, and may be important for human health and longevity [37,38]. Thus, GABP/NRF2 may coordinate the cytosolic and mitochondrial protein synthesis. Impairment of mitochondrial function, probably caused by ROS, may be associated with age-related disorders such as type 2 diabetes [39] and Alzheimer's disease [40]. Mitochondrial turnover is higher in young individuals compared to middle-aged and older subjects [41], and genes involved in renewal of mitochondria are downregulated in old mice [42]. Increased mitochondrial number has also been associated with the life-prolonging effects of exercise as well as energy restriction in rats [43,44]. The effect of energy restriction on longevity in *Caenorhabditis elegans *is linked to genes that increase mitochondrial activity throughout the body [45,46]. In addition, resveratrol reversed the decline of hepatic mitochondrial number in mice fed a high-energy diet [47]. In this study, we also found genes sharing regulatory motifs for another transcription factor controlling mitochondrial gene transcription, Yin Yang 1 (YY1) [48], to be upregulated in both intervention groups. The antioxidant-rich diets may thus offer a health benefit regarding chronic age-related diseases by influencing mitochondrial biogenesis via induction of GABP/NRF2 and YY1 target genes.

The antioxidant-rich diet presented here is not only rich in antioxidants, but provided nutrients such as monounsaturated and polyunsaturated lipids and folate (magnesium and iron). The effects on gene expression observed in this intervention study may therefore be attributed not only to phytochemicals but also to other dietary compounds acting via several different mechanisms. However, as similar effects are exerted by the kiwifruit diet, it is plausible to suggest that the observed modulation is mediated mainly by plant-based compounds. Another important aspect is that we have utilised blood cells to assess the effect of the interventions on gene expression. Thus, all the cellular processes found to be regulated by the diets may be of importance for immune function.

## Conclusions

This human dietary intervention is the first to investigate the influences of antioxidant-rich diets on gene expression in whole blood. We observe that gene sets related to DNA repair, hypoxia, apoptosis and immune processes are significantly upregulated by a complex antioxidant-rich diet or by kiwifruits. Our results suggest that the beneficial health effects of a plant-based diet may involve modulation of stress- and defence-related gene expression important for maintenance of cellular functions. Whether this effect is caused by modulation of redox homeostasis or via other mechanisms needs further investigation. The reported results may contribute to the development of public nutritional advice on antioxidant intake in aspects of prevention of oxidative stress-related diseases and subsequent healthy aging, although the conclusions are preliminary.

## Abbreviations

AA: ascorbic acid; AhR: aryl hydrocarbon receptor; AhR/ARNT(HIF1B): AhR nuclear translocator; ALAT: alanine-aminotransferase; ANOVA: analysis of variance; ARE/EpRE: antioxidant responsive element; BLAST: basic local alignment search tool; BMI: body mass index; CKM: creatine kinase muscle; CTNND1: catenin (cadherin-associated protein)-δ 1; CUTL1L: cutlike 1; CVD: cardiovascular diseases; DOPAC: 3,4-dihydroxyphenylacetic acid; ELK1: ETS oncogene family; ETS: E-26. FDR: false discovery rate; FRAP: ferric reducing/antioxidant power assay; GK2: glycerol kinase 2; GO: gene ontology; GPR173: G protein-coupled receptor 173; GSEA: gene set enrichment analysis; HIF1A: hypoxia-inducible factor 1; HUGO: Human Genome Organisation; LIPE: lipase; hormone-sensitive; MIAME: Minimum Information About a Microarray Experiment; NHEDC1: Na^+^/H^+ ^exchanger domain containing 1; MUFA: monounsaturated fatty acids; NR0B1: nuclear receptor subfamily 0, group B, member 1; NRF2/GABP: nuclear respiratory factor 2/GA binding protein transcription factor; Nrf2/NFE2L2: nuclear factor erythroid 2-related factor 2; NTN3: immunoglobulin kappa light chain variable region; OR2F1: olfactory receptor, family 2, subfamily F, member 1; PNA: peptide nucleic acid; PPARγ: peroxisome proliferator-activated receptor-γ; PUFA: polyunsaturated fatty acids; SFA: saturated fatty acids.

## Competing interests

The following authors declare no competing interest; SKB, MCM, MT, MH, SHT, AK, IE MS, IS, JØM, AKD, PL, HA, ST and AC. RB and CAD have an interest in Bioindex AS and Vitas AS, and RB has an interest in Cgene AS. Bioindex and Cgene were established by Birkeland Innovation, the technology transfer office at the University of Oslo, while Vitas was established by Oslo Innovation Center.

## Authors' contributions

RB and SKB formulated the present hypothesis. SKB, MCM, MT, MH, AK, MS, IS, JØM, AKD, PL, CAD, HA, ST, AC, RB contributed to the clinical study design, intervention, and sample collection. MCM was responsible for RNA isolation. SKB was responsible for quality control of samples and responsible for the statistical analysis, together with MT and MH. SHT did the hybridization to the arrays. AK was responsible for analysis of plasma carotenoids. IE was responsible for the analysis of polyphenols in plasma. SKB and MCM were responsible for interpreting the results and drafting the manuscript. All authors approved the final version before submission.

## Pre-publication history

The pre-publication history for this paper can be accessed here:

http://www.biomedcentral.com/1741-7015/8/54/prepub

## Supplementary Material

Additional file 1 Document S1: A full description of the methods for plasma antioxidant analysis, with references. Click here for file

Additional file 2 **Figure S1: The figure obtained using Metacore illustrates the leading edge (LE) genes (red bars) (contributing to the significance of the upregulated DNA and repair gene sets in GSEA) in the Nucleotide excision repair (NER) pathway.** Red bars indicate LE genes from (1) comparing antioxidant-rich diet group to controls and (2) from comparing kiwifruit diet to controls. Click here for file

Additional file 3 **Figure S2: The figure obtained using Metacore illustrates the leading edge (LE) genes (red bars) (contributing to the significance of the upregulated DNA and repair gene sets in GSEA) represented in the Mismatch repair pathway.** Red bars indicate LE genes from (1) comparing antioxidant-rich diet group to controls and (2) from comparing kiwifruit diet to controls. Click here for file

Additional file 4 **Figure S3: The figure obtained using Metacore illustrates the leading edge (LE) genes (red bars) (contributing to the significance of the upregulated DNA and repair gene sets in GSEA) in the response to double -strand breaks-pathway.** Red bars indicate LE genes from (1) comparing antioxidant-rich diet group to controls and (2) from comparing kiwifruit diet to controls. Click here for file

Additional file 5 **Table S1: Overview of the gene sets in the stress relevant gene set collections that were upregulated in the antioxidant-rich diet group when compared to controls (GSEA analysis)**. A brief description of each gene set is included with PubMed ID reference. Click here for file

Additional file 6 **Table S2: Overview of the gene sets in the stress relevant gene set collections that were upregulated in the kiwifruit group when compared to controls (GSEA analysis).** A brief description of each gene set is included with PubMed ID reference. Click here for file

Additional file 7 Table S3: Gene sets in the C3 TFT collection regulated in the antioxidant-rich diet group and kiwifruit group when compared to controls Click here for file

Additional file 8 Table S4: Gene transcripts upregulated in the antioxidant-rich diet -group vs. controls (LIMMA, p < 5%) Click here for file

Additional file 9 Table S5: Gene transcripts upregulated in the kiwifruit-group vs. controls (LIMMA, p < 5%) Click here for file

Additional file 10 Table S6: Gene transcripts downregulated in the antioxidant-rich diet-group vs. controls (LIMMA, p < 5%) Click here for file

Additional file 11 Table S7: Gene transcripts downregulated in the kiwifruit -group vs. controls (LIMMA, p < 5%) Click here for file

Additional file 12 Table S8: Biological processes significantly enriched among the upregulated gene transcripts in the antioxidant-rich diet group. Click here for file

Additional file 13 Table S9: Biological processes significantly enriched among the downregulated gene transcripts in the antioxidant-rich diet group. Click here for file

Additional file 14 Table S10: Biological processes significantly enriched among the upregulated gene transcripts in the kiwifruit group. Click here for file

Additional file 15 Table S11: Biological processes significantly enriched among the downregulated gene transcripts in the kiwifruit group. Click here for file
