# Supplementary material for: Blood cell gene expression associated with cellular stress defense is modulated by antioxidant-rich food in a randomised controlled clinical trial of male smokers
Source: BMC Med. 2010 Sep 16;8:54. doi: 10.1186/1741-7015-8-54 (PMC2955589; doi:10.1186/1741-7015-8-54)
Supplement: Additional file 1 — Document S1: A full description of the methods for plasma antioxidant analysis, with references. [file 1741-7015-8-54-S1.DOC]

## Methods

***Plasma carotenoid analysis***

The carotenoids lutein, zeaxanthin, β-kryptoxanthin, α-carotene, β-carotene and lycopene were determined in plasma by HPLC. Proteins were precipitated and removed by the addition of a 4.5 volume of isopropanol followed by centrifugation at 3.000 g at 4 °C for 15 min. The internal standard astaxanthin was added with the isopropanol. Twenty five µL of the clear supernatant were used for analysis. The mobile phases consisted of A: 20 % water and 24 % acetone in ethanol and B: acetone. The gradient conditions were as follows: From 2 to 100 % B within 20 min, followed by 100 % B for 15 min. Detection was performed at 453 nm using a variable wavelength detector. Plasma calibrators quantified against the NIST 968c SRM were used as standards.
