# Supplementary material for: Blood cell gene expression associated with cellular stress defense is modulated by antioxidant-rich food in a randomised controlled clinical trial of male smokers
Source: BMC Med. 2010 Sep 16;8:54. doi: 10.1186/1741-7015-8-54 (PMC2955589; doi:10.1186/1741-7015-8-54)
Supplement: Additional file 2 — Figure S1: The figure obtained using Metacore illustrates the leading edge (LE) genes (red bars) (contributing to the significance of the upregulated DNA and repair gene sets in GSEA) in the Nucleotide excision repair (NER) pathway. Red bars indicate LE genes from (1) comparing antioxidant-rich diet group to controls and (2) from comparing kiwifruit diet to controls. [file 1741-7015-8-54-S2.PPT]

## Slide 1
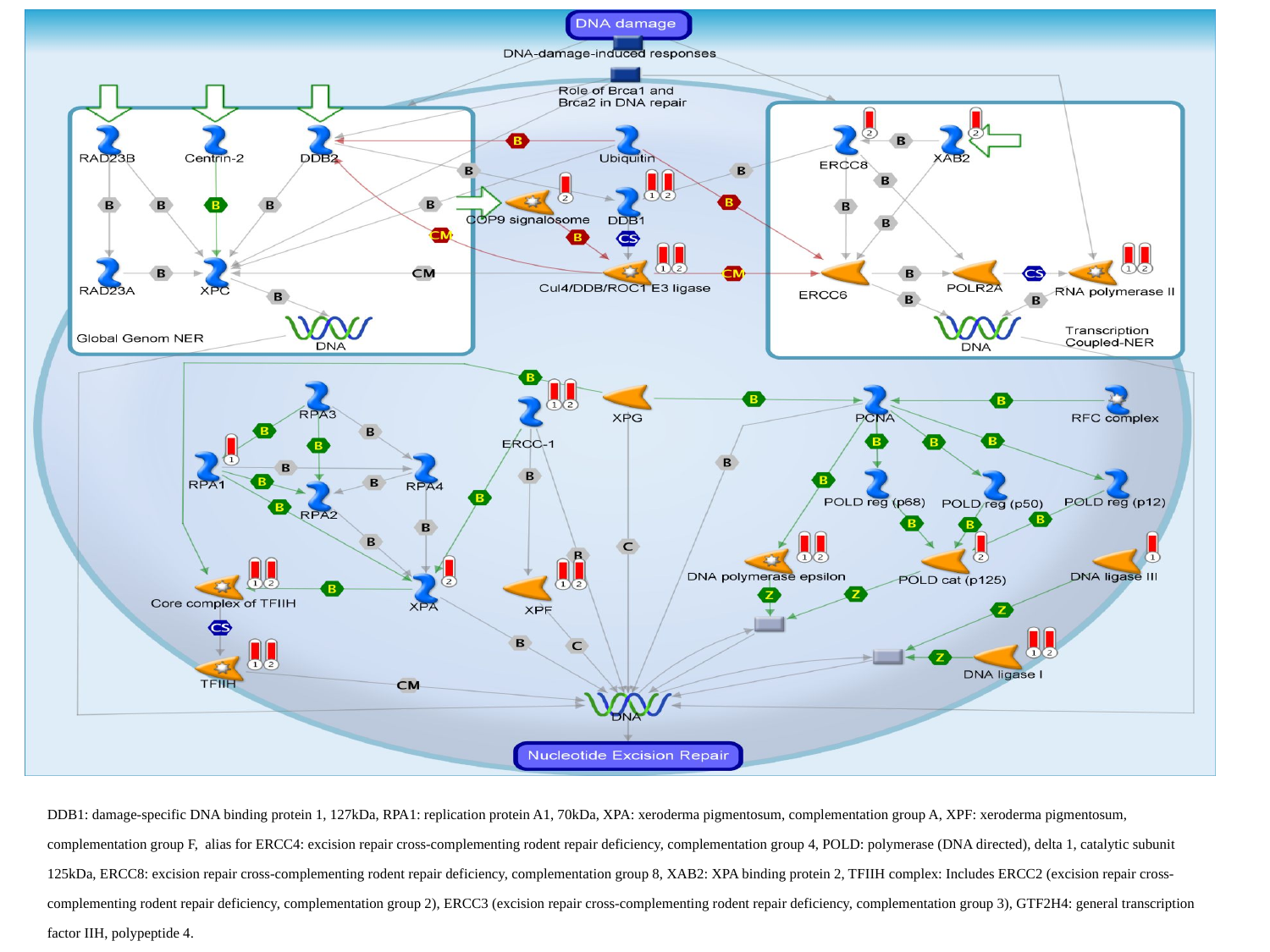

DDB1: damage-specific DNA binding protein 1, 127kDa, RPA1: replication protein A1, 70kDa, XPA: xeroderma pigmentosum, complementation group A, XPF: xeroderma pigmentosum, complementation group F, alias for ERCC4: excision repair cross-complementing rodent repair deficiency, complementation group 4, POLD: polymerase (DNA directed), delta 1, catalytic subunit 125kDa, ERCC8: excision repair cross-complementing rodent repair deficiency, complementation group 8, XAB2: XPA binding protein 2, TFIIH complex: Includes ERCC2 (excision repair cross-complementing rodent repair deficiency, complementation group 2), ERCC3 (excision repair cross-complementing rodent repair deficiency, complementation group 3), GTF2H4: general transcription factor IIH, polypeptide 4.
