# Supplementary material for: Blood cell gene expression associated with cellular stress defense is modulated by antioxidant-rich food in a randomised controlled clinical trial of male smokers
Source: BMC Med. 2010 Sep 16;8:54. doi: 10.1186/1741-7015-8-54 (PMC2955589; doi:10.1186/1741-7015-8-54)
Supplement: Additional file 3 — Figure S2: The figure obtained using Metacore illustrates the leading edge (LE) genes (red bars) (contributing to the significance of the upregulated DNA and repair gene sets in GSEA) represented in the Mismatch repair pathway. Red bars indicate LE genes from (1) comparing antioxidant-rich diet group to controls and (2) from comparing kiwifruit diet to controls. [file 1741-7015-8-54-S3.PPT]

## Slide 1
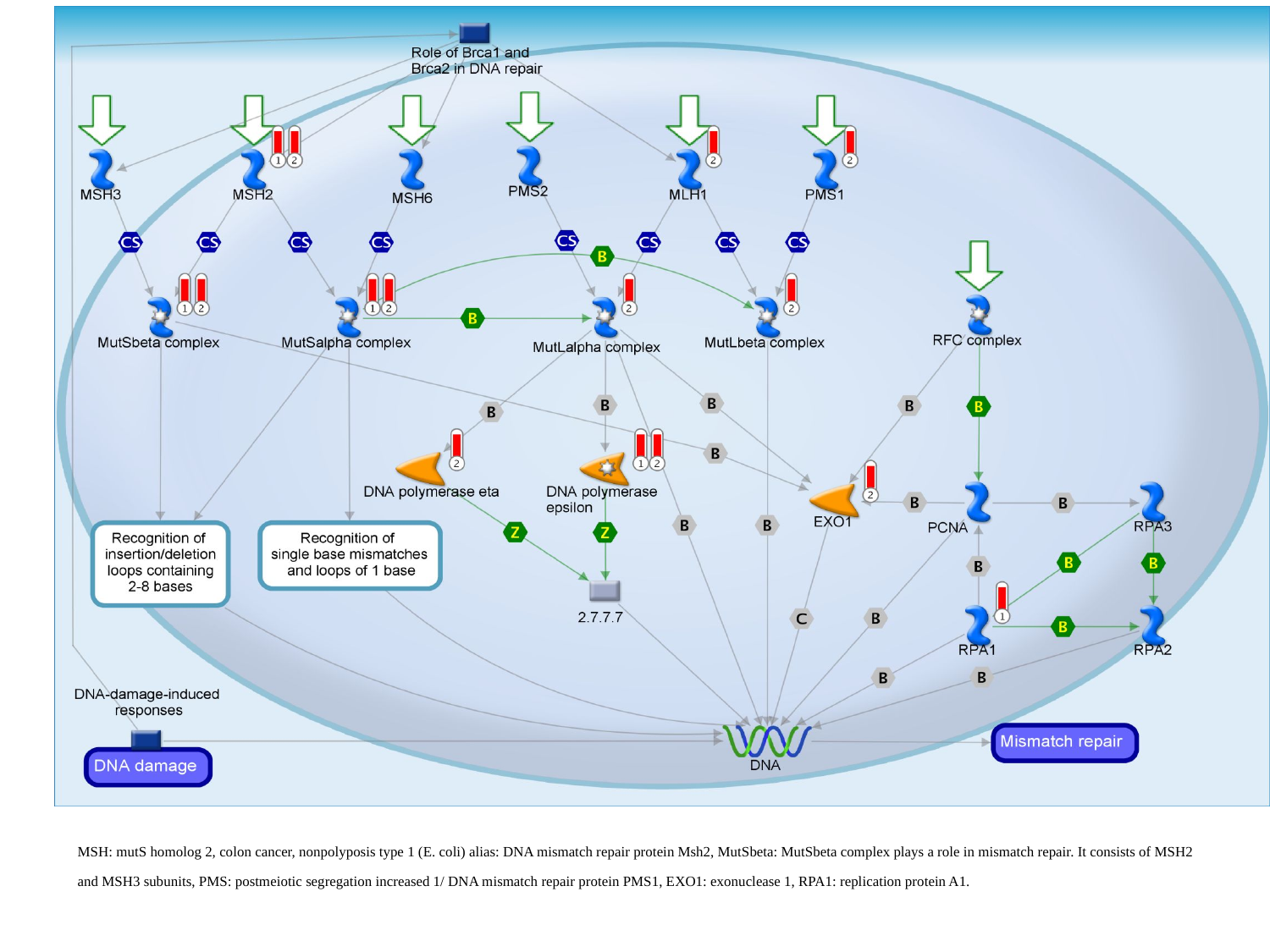

MSH: mutS homolog 2, colon cancer, nonpolyposis type 1 (E. coli) alias: DNA mismatch repair protein Msh2, MutSbeta: MutSbeta complex plays a role in mismatch repair. It consists of MSH2 and MSH3 subunits, PMS: postmeiotic segregation increased 1/ DNA mismatch repair protein PMS1, EXO1: exonuclease 1, RPA1: replication protein A1.
