# Supplementary material for: Blood cell gene expression associated with cellular stress defense is modulated by antioxidant-rich food in a randomised controlled clinical trial of male smokers
Source: BMC Med. 2010 Sep 16;8:54. doi: 10.1186/1741-7015-8-54 (PMC2955589; doi:10.1186/1741-7015-8-54)
Supplement: Additional file 4 — Figure S3: The figure obtained using Metacore illustrates the leading edge (LE) genes (red bars) (contributing to the significance of the upregulated DNA and repair gene sets in GSEA) in the response to double -strand breaks-pathway. Red bars indicate LE genes from (1) comparing antioxidant-rich diet group to controls and (2) from comparing kiwifruit diet to controls. [file 1741-7015-8-54-S4.PPT]

## Slide 1
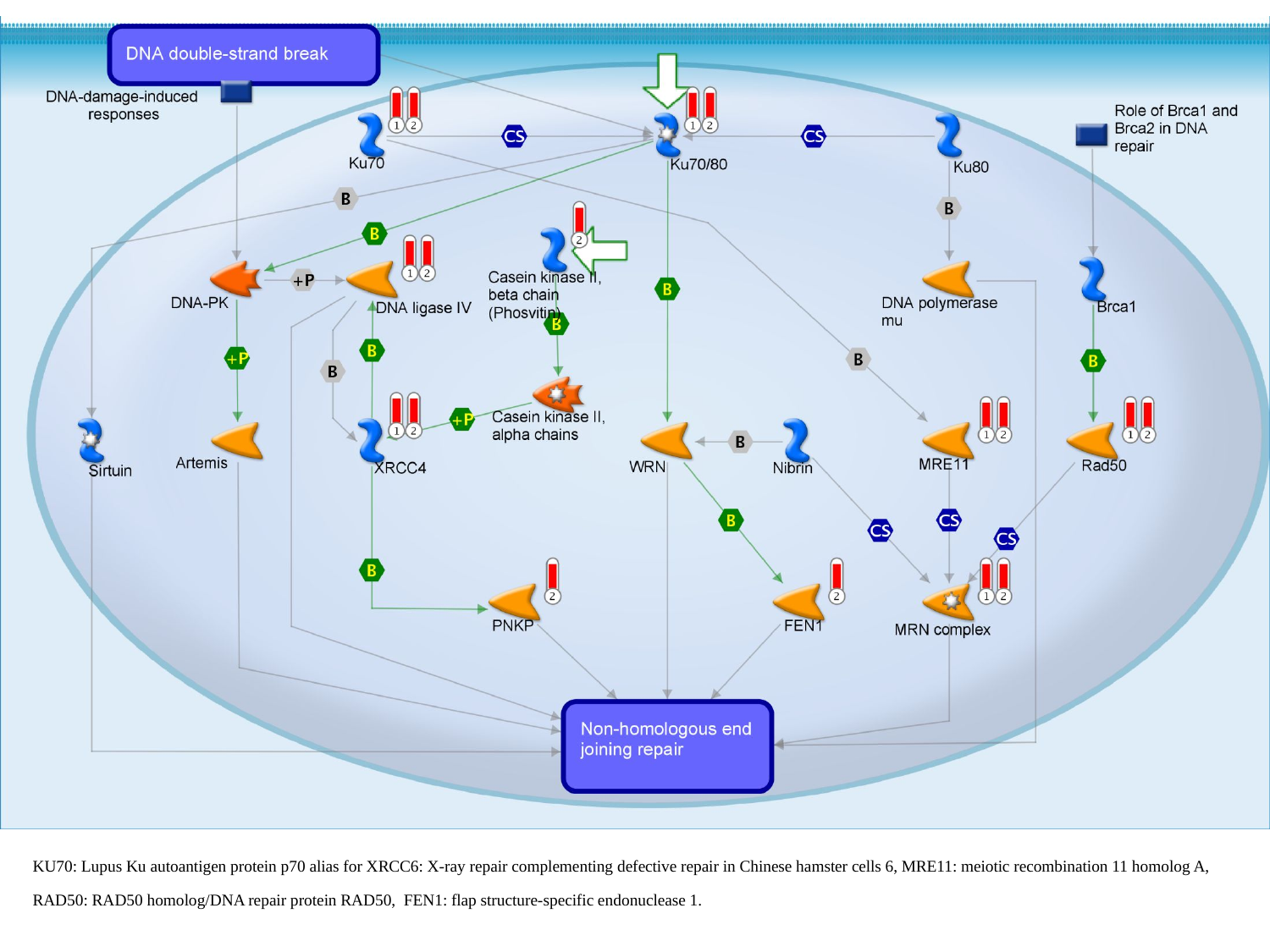

KU70: Lupus Ku autoantigen protein p70 alias for XRCC6: X-ray repair complementing defective repair in Chinese hamster cells 6, MRE11: meiotic recombination 11 homolog A, RAD50: RAD50 homolog/DNA repair protein RAD50, FEN1: flap structure-specific endonuclease 1.
